# Crash Course in Orthopaedics: Development and Evaluation of a 12-Week Online Trauma and Orthopaedics Teaching Program for Medical Students During the COVID-19 Pandemic

**DOI:** 10.7759/cureus.28628

**Published:** 2022-08-31

**Authors:** Catherine James, Karen Chui, Alex Brown, Arwel Poacher, Claire Carpenter, Edwin Jesudason

**Affiliations:** 1 Orthopaedics, Cardiff & Vale University Health Board, Cardiff, GBR; 2 Orthopaedic Surgery, Royal National Orthopaedic Hospital, Stanmore, GBR; 3 Orthopaedic Surgery, Swansea Bay University Health Board, Swansea, GBR; 4 Orthopedic Surgery, Betsi Cadwaladr University Health Board, Bangor, GBR

**Keywords:** webinar series, conducting a webinar, online medical education, virtual learning environment, virtual learning, online learning, covid-19, orthopaedics, webinar

## Abstract

Introduction

The COVID-19 pandemic caused significant disruption in clinical placements of medical students in the United Kingdom (UK), including trauma and orthopaedic surgery (T&O) rotations. Based on the British Orthopaedic Association (BOA) undergraduate syllabus, a 12-week online teaching program was designed to supplement T&O teaching for medical students across the UK while lockdown and social-distancing restrictions were in place. This study aims to describe the process of designing an online teaching program, evaluate the effectiveness of online education, explore medical student perceptions of the virtual learning environment, and report the lessons learned from this 12-week online program.

Methods

The “Crash Course in Orthopaedics” consisted of 12 webinars, with topics covering a range of acute and chronic T&O conditions, and was delivered through the online platform Zoom. Attendees were invited to complete a post-course questionnaire retrospectively and the results were used in this study. Qualitative data was assessed using thematic analysis. Quantitative data were presented as descriptive statistics.

Results

The webinar series was attended by approximately 5150 participants, with the largest demographic group being clinical medical students (49%). Results from the survey revealed three broad themes which were:

1). Interactivity: question + answer (Q+A), multiple choice questions (MCQs), online tools

2). Content: case examples, orthopaedic examinations, objective structure clinical examination (OSCE) tips

3). Accessibility: slides, recordings, duration of the session.

Our study found that the online teaching program improved students’ clinical knowledge of T&O and they found learning through interactive methods such as polls, the chat function on zoom, and case-based discussions to be most useful. Also, from the results of this study, a guide on “How to Run a Successful Webinar Series for Medical Students” was developed.

Conclusion

Online webinars effectively supplement T&O teaching and experience for medical students whose T&O placements were disrupted during the COVID-19 pandemic. The results will be a helpful guide to those planning medical education webinars in the future.

## Introduction

In March 2020, the World Health Organisation (WHO) declared the novel coronavirus (COVID-19) outbreak a global pandemic [1]. To reduce the spread of the virus, drastic measures were taken, which included a nationwide lockdown in the United Kingdom (UK).

During the pandemic, medical schools in the UK suspended hospital-based clinical placements and face-to-face lectures and seminars temporarily. This had a detrimental impact on the traditional delivery of medical education, particularly affecting medical students’ experience and knowledge of specialties already afforded less time by medical schools, such as trauma and orthopaedic surgery (T&O). Undergraduate T&O teaching was limited in the UK even prior to the COVID-19 pandemic, with the mean duration of clinical placement in T&O being only 2.5 weeks [2]. In addition, despite one-third of primary care consultations relating to musculoskeletal problems, approximately 20% of final-year medical students reported they never had a placement in T&O [3].

Medical schools were obliged to find alternative ways to deliver teaching and most adapted the syllabus material into a deliverable online format, enabling the continuation of teaching and minimizing the disruption in studies [4]. As T&O teaching was already limited prior to the pandemic, there was a need for additional T&O educational materials to be delivered by alternative teaching methods [2]. 

Studies have demonstrated virtual teaching to be as effective, if not more effective than offline learning [5]. Students report the benefits of online education as improved availability and flexibility; reduction in travel time and cost; a more comfortable learning environment; and the ability to be interactive and ask questions in a safe space. Distractions, poor internet connection, and timing of tutorials were listed as barriers to online learning [6]. During the COVID-19 pandemic, there was a significant increase in the average amount of time students spent learning online per week [6]. With the pandemic accelerating the advent of virtual learning, there is a need to understand how to design and deliver effective online learning platforms and formats.

This cross-sectional observational study aimed to develop, implement, and evaluate a 12-week online teaching program “Crash Course in Orthopaedics” during the COVID-19 pandemic. The main objectives were to evaluate the effectiveness of the online teaching program; explore medical student perceptions of virtual T&O teaching; and understand aspects of virtual teaching students found most useful, by using a post-course evaluation questionnaire. This will then provide guidance on improving future online educational materials for medical students.

## Materials and methods

A 12-week webinar series titled “Crash Course in Orthopaedics” based on the British Orthopaedic Association (BOA) Undergraduate Syllabus [7], which outlines the expected T&O knowledge of medical students upon graduation from medical school in the UK, was designed to supplement disrupted T&O teaching and experience for medical students during the pandemic. The structure of the series is outlined in Table 1.

**Table 1:** Structure of the webinar series

Webinar Number	Topic	Speaker
1	The Limping Child	Consultant surgeon
2	Musculoskeletal History Taking	Final year medical student
3	Musculoskeletal X-ray Interpretation	Consultant surgeon
4	Hip Fractures	Academic foundation doctor
5	Orthopaedic Emergencies	Final year medical student
6	The Shoulder	Consultant surgeon
7	The Hand	Consultant surgeon
8	The Spine	Consultant surgeon
9	The Knee	Consultant surgeon
10	The Foot & Ankle	Consultant surgeon
11	The Hip	Consultant surgeon
12	Surviving Your First On-call	Core surgical trainee

The webinars discussed common acute and chronic T&O conditions, as outlined in the BOA undergraduate syllabus, including the etiology, risk factors, pathophysiology, investigations, differential diagnoses, and management of these conditions. A combination of didactic teaching, case-based discussions, and multiple choice questions (MCQs) were used during the webinars. 

The webinar series was advertised four weeks prior on social media platforms, Facebook and Twitter, and via email to an administrator at each medical school in the UK. None of the online advertisements required payment. Interested students registered online to attend the webinars for free.

The webinars were delivered live using the online platform Zoom, which had a capacity of 1,000 participants. The teaching slides were created with Microsoft PowerPoint or Keynote, depending on speaker preference. Teaching slides were shared with participants during the session using the ‘Share Screen’ function on Zoom.

Participants were asked to be muted with cameras turned off to minimize disruption during the webinars. Participants were encouraged to comment, ask, and answer questions using the ‘Chat’ and ‘Poll’ functions on Zoom. The speakers also used interactive activities during the webinar to enable engagement from the participants.

The webinars were 45 to 90 minutes in duration and held after-work hours, allowing students in clinical years to attend. The series was delivered over a 12-week period, with one webinar per week. Invitations for speakers were sent out to the local orthopaedic department, with the webinars then being delivered by a variety of tutors, from final year medical students to consultant orthopaedic surgeons as outlined in Table 1.

Participants were invited to complete post-course feedback questionnaires created and disseminated on Google forms after each webinar. The questionnaire was developed to evaluate the effectiveness of the teaching and to assess what aspects of virtual teaching students found most useful. The questionnaire included quantitative five-point Likert scales and free text comments to enable qualitative evaluation of the webinars. Qualitative data analysis was performed using a thematic approach, whereby inductive analysis was conducted in which common themes were extracted from the data. [8]. Quantitative post hoc analysis included descriptive statistics from the five-point Likert scales. Informed consent was obtained by participants clicking “Yes” before proceeding with the feedback questionnaire.

## Results

**Figure 1:**
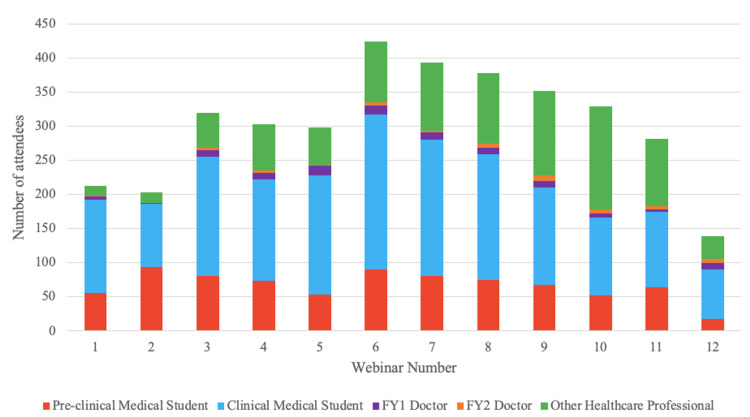
Graph to show the number of attendees at each webinar

The webinar series was attended by a total of 5142 participants, with an average of 303 participants per webinar. 3635 of 5142 participants (71%) completed the post-course questionnaire. Participation in the webinar series and completion of the questionnaire was voluntary, with no financial compensation for participating. ‘The Shoulder’ webinar (Webinar 6) had the highest number of participants at 424 and ‘Surviving your 1st on-call’ (Webinar 12) was the least attended webinar with 139 participants (Figure 1).

Demographics of Attendees

Medical students in their clinical years of training were the largest group that attended the webinars (49%), followed by other health care professionals (25%) and pre-clinical medical students (22%) (Table 2). Other healthcare professionals included: physiotherapists, general practitioners, paramedics, nurses, physician associates, and occupational therapists.

**Table 2:** Level of training of participants in attendance at the webinar series

Level of Training	n	%
Pre-clinical medical student	802	22
Clinical medical student	1779	49
Foundation year 1 doctor	102	3
Foundation year 2 doctor	48	1
Other health care professionals	904	25
Total	3635	100

Most medical students were from UK universities, with Norwich Medical School, England, having the highest number of attendees, followed by Cardiff University, Wales. The webinar series was also attended by international medical students, including participants from medical schools in 61 other countries worldwide (Table 3). International participants discovered the webinar series through social media only.** **

**Table 3:** List of countries of origins of webinar series participants

Country	n
United Kingdom	2259
Malta	239
Romania	93
Poland	84
Latvia	81
South Africa	65
Bulgaria	55
North Macedonia	53
Malaysia	43
Estonia	40
Pakistan	40
Georgia	37
Greece	34
Germany	33
Bosnia and Herzegovina	31
Slovakia	29
Egypt	28
Ireland	23
China	22
Canada	21
India	21
Lithuania	21
Montenegro	20
Croatia	19
Sri Lanka	19
Cyprus	17
Albania	16
Hungary	16
Sweden	16
Australia	14
Mauritius	12
USA	12
Czech Republic	11
Austria	9
Italy	9
Philippines	8
Netherlands	7
Norway	7
Myanmar	6
Belgium	5
Libya	5
Portugal	5
Ukraine	5
Belgrade	4
Denmark	4
Saudi Arabia	4
Turkey	4
Bangladesh	3
Finland	3
Peru	3
Switzerland	3
Brazil	2
Jordan	2
Kenya	2
Morocco	2
New Zealand	2
Panama	2
Crete	1
Ethiopia	1
Malawi	1
Trinidad and Tobago	1
Yemen	1
Total	3635

Advertisement

The webinar series was advertised through social media and emails. 84% of attendees discovered the webinar series through platforms such as Facebook and Twitter. Table 4 outlines how participants discovered the webinar series. Responses to this question were only collected during the second half of the series, therefore the number of responses was fewer.

**Table 4:** How participants discovered the webinar series

Type of Advertisement	n	%
Facebook	1900	83
Twitter	24	1
Through their university	243	11
Recommended by a friend/ colleague	116	5
Attendance at previous webinar	15	1
Total	2298	100

Post-course feedback questionnaire

**Figure 2:**
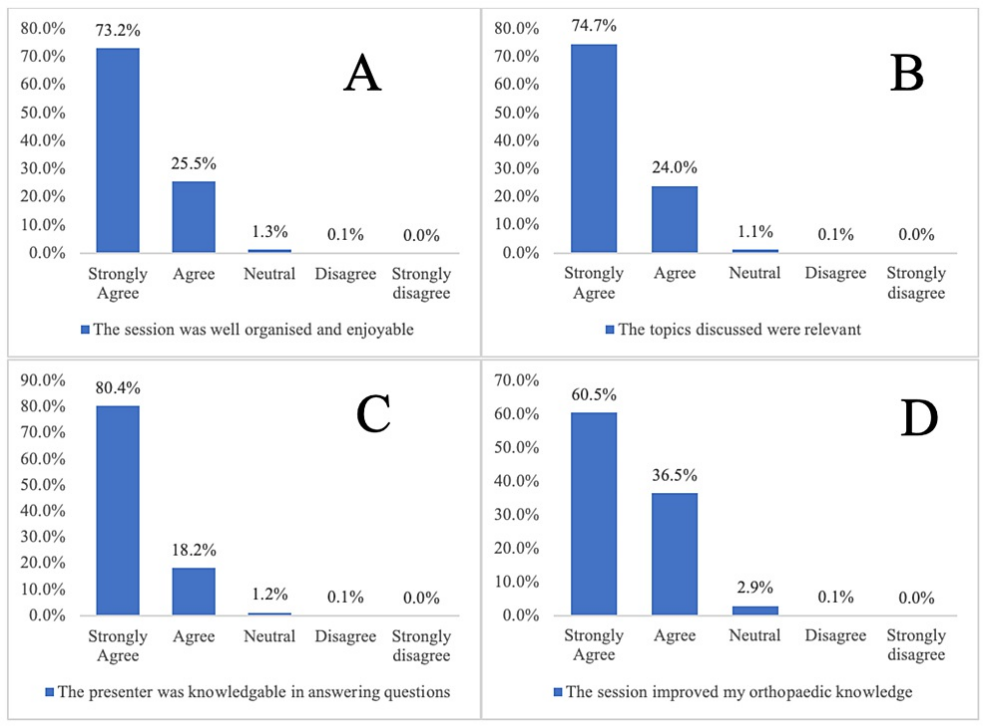
Post-course feedback questionnaire responses to A. Organisation and enjoyability of the webinar series, B. Clinical relevance of the webinar sessions, C. Orthopaedic experience of presenter, and D. Improvement of participant orthopaedic knowledge.

Participants were asked to evaluate the webinar sessions using five-point Likert scales. Seventy-three percent of participants ‘Strongly Agree’ and 25.5% ‘Agree’ that the webinars were well organized and enjoyable (Figure 2A). When asked ‘The topics discussed were clinically relevant’, 74.7% of participants ‘Strongly agree’ and 24.0% ‘Agree’ (Figure 2B). Regarding the orthopaedic experience of the presenters, 80.4% ‘Strongly Agree’ and 18.2% ‘Agree’ that the presenter was knowledgeable in answering questions (Figure 2C). Of those who responded, 60.5% ‘Strongly Agree’ and 36.5% ‘Agree’ that the sessions improved their orthopaedic knowledge (Figure 2D).

Qualitative data analysis revealed three main themes. The first related to the interactivity of the webinars, the second focused on the content and topics covered across the series, and the third was related to the accessibility of the webinar series (Table 5).

**Table 5:** Themes and subthemes emerging from the study OSCE: objective structure clinical examination

Theme	Subthemes
Interactivity	Question + answer, Multiple choice questions, Online tools
Content	Case examples, Orthopaedic examinations, OSCE tips
Accessibility	Slides, Recordings, Session duration

Theme one - interactivity

Each webinar employed various interactive tools, such as the ‘Poll’ and ‘Chat’ functions. The more interactive webinars received comments such as “the inclusion of interactive elements helped to keep the audience engaged”. Whereas those with less interactivity had feedback such as “more interaction would be appreciated”. The subthemes relating to interactivity included potential methods to improve participant engagement during the webinar, such as questions and answer (Q+A) sessions, MCQs, and using online tools such as Kahoot.

Feedback such as “The Q+A session at the end made it interactive” and “appreciate the opportunity to ask questions” demonstrated the positive aspect of including a Q+A session. However, participants had differing opinions on the optimal time during the webinar for the speaker to answer participants’ questions, with some preferring the speaker to pause after each topic to answer questions and others preferring the speaker to answer questions at the end of the webinar because “it causes a delay and sometimes the speaker may answer the question later during the session”.

When participants were asked “What did you like about the teaching session?” participants frequently responded with the use of multiple-choice questions (MCQs) [or single best answers (SBAs)]. If MCQs were included in the webinar, participants would state they wanted “more MCQs throughout the webinar”, and similarly, if the speaker did not use MCQs during the session, participants would provide feedback such as “future use of MCQs to help with engagement and understanding”.

The in-application ‘Poll’ function on Zoom was the only online tool used by some speakers during the webinar series, and participants “enjoyed the chance to take part by answering polls”. On analysis of the feedback, some participants suggested alternative online tools such as Mentimeter or Kahoot, to “further encourage audience participation and engagement” by enabling participants to answer questions in a game-like format.

Theme two - content

The content of the teaching program was based on the BOA undergraduate syllabus [7], and speakers were provided with a list of topics to include in their sessions. However, the structure of each webinar and the method of teaching were at the discretion of the individual speaker. Three subthemes appeared relating to the content of the webinars, these were: case examples, orthopaedic examinations, and objective structure clinical examination (OSCE) tips.

When asked, “What did you like about the teaching session?”, participants commonly answered “the use of clinical cases”, with comments such as “it was very useful to go through cases and apply knowledge to a clinical scenario”. However, for the webinars that mainly focused on case examples, participants commented that the session could be improved by including “some theoretical teaching” or “basics” before going through the clinical cases, in addition to providing “a summary slide at the end of each case with an overview of the key points”.

Due to time constraints and the challenges of teaching clinical examination skills virtually, most webinars focused on clinical knowledge rather than orthopaedic examination skills. Analysis of the feedback revealed comments relating to the inclusion of orthopaedic examination skills in future webinars, such as “use videos in slides to show examinations” or “live examination to demonstrate orthopaedic examinations”.

Most medical schools employ OSCEs to assess clinical performance. Following the thematic analysis of the feedback collected, the third subtheme related to content was OSCE tips. Participants suggested improvements could include discussions around advice and tips for approaching and passing OSCEs, with comments such as “inclusion of OSCE style questions/scenarios” and “more tips for exams/OSCEs”.

Theme three - accessibility

The final theme that emerged was the accessibility of the webinar series with three subthemes: presentation slides, recordings of the webinars, and the session duration.

Feedback analysis found that students preferred for the slides to be made available prior to the webinar. For example, “it would be great if the slides could be sent prior to the talk so students can annotate them during the session”. In addition, many students also requested access to the slides after the webinar. Other comments were related to the presentation and format of the slides with feedback such as: “the slides could have slightly more detail in them”, “maybe make the slides a little neater”, and “increase the size of the font used for the slides” and “slightly larger images on the slides”.

The second subtheme related to access to recordings of the webinars. Recordings of webinars enabled students to replay the webinar, review the session in their own time and make additional notes as required. In addition, it allowed students who could not attend the session live due to other commitments to replay the webinar later. Comments related to the recording of the webinars included: “I would like to receive a recording of the session so that I can re-watch some of the explanations”, “A recording of the lecture to go over in my own time” and “Recording the session for people who are not available for the live webinar”.

Regarding the session duration, each webinar was aimed for a duration of 45 minutes. However, inevitably some overran with the longest session being 90 minutes. Students were positive when the webinar ran to time with comments such as “the session ran to time which was great”, but also commented if the session overran with “please keep to time”. Students expressed positive enjoyment when there was time left during the webinar for questions. There were many differing comments regarding the session length and time, with some students preferring shorter (as short as 30 minutes) and others preferring longer sessions (up to two hours). The most favored duration was one hour compromising 45 minutes for presenting and 15 minutes in the end for questions, in addition to advising participants at the start how long the session is expected to last.

## Discussion

The lessons learned from this study have demonstrated the key components of a successful webinar series. Interactivity is imperative to an enjoyable and engaging webinar. Actively involving participants leads to an increased interest in the topic and better retention of knowledge and skills [9]. The use of interactive pedagogic tools (IPT), such as Kahoot or Mentimeter, is well-received by participants and helps with their learning and understanding of important concepts [10]. Additionally, the use of MCQs, polls and open questions throughout the webinar enables audience engagement and increases their enjoyment. It is important that the speaker is familiar with the online platform and the interactive tools they intend to use, to prevent any delays and interruptions to the webinar.

During webinars, participants should be given the opportunity to ask questions to fill their knowledge gaps and seek clarification for aspects they misunderstood. Ideally, at the start of the webinar, the speaker should clarify their preferences with regard to the timing of questions. Some speakers may prefer for participants to ask questions throughout, whereas others may prefer to leave questions until the end of the webinar, as they may answer the question later during the session. For webinars with approximately 30 attendees, it may be suitable for the participants to unmute themselves and ask the question aloud. However, for webinars where there are more than 30 attendees, this may cause background noise and poor audio quality so typing questions in the chat function would be the preferred method [11]. This method also enables participants who are less comfortable speaking up and those whose first language is not the one used for the webinar, to feel more comfortable asking their questions [12].

The main priority for many medical students is passing exams and graduating from medical school. Therefore, understandably any exam practice and advice they can be given is gratefully received. Studies show the inclusion of MCQs is beneficial in allowing students to practice exam techniques and clinical reasoning [13]. However, creating MCQs is a skill, and the questions asked should aim to reflect the webinar’s learning objectives [10].

A key criticism from participants was the absence of clinical examination content. A suggestion would be doing a separate OSCE style-focused webinar plus online mock exams, in addition to the theoretical knowledge-focused webinars. It has been demonstrated that students feel they benefit from video examples of the examinations [14]. There are resources available online, but these may face copyright issues, or may not illustrate the specific point being raised. However, speakers could include such resources as additional learning materials for participants. Confidentiality is often a reason why speakers do not prepare video examples with their own patients. In addition, there may be variability in video and audio quality that impacts the effectiveness of the videos, especially when playing videos through a webinar platform such as Zoom.

Most speakers (92%) used Microsoft PowerPoint to create presentation slides for the webinar. The feedback showed potential areas of improvement in relation to the clarity and quality of the slides. Speakers should aim to use size 32 font and include images to supplement the text. With some webinar platforms, the attendees may only be able to see the slides and not the speaker, therefore the slides need to be clear and engaging. Additionally, the use of acronyms or abbreviations, without explanation, should be avoided as participants may not be familiar with them [11]. Supplementary text and comments in the notes section on PowerPoint could be provided to participants afterward the webinar, to prevent overloading slides with text which may distract attention away from the speaker.

The results show there is a strong demand for recordings of the webinars to be made available to the participants. This allows participants to replay the webinar to review areas they do not immediately understand and those who were unable to attend live to participate. However, recordings of the webinars raise issues surrounding copyright and confidentiality. Methods to address these include inserting attribution links to authors of stock images or the source URL for google images. General Medical Council (GMC) guidance allows anonymized patient data to be used for the purposes of teaching without patient consent, although access to the file would need to be secured to ensure it is used only for this purpose [15].

Previous research suggests the optimum length for traditional didactic teaching is 15-30 minutes, however, this is not feasible for a complex topic [16]. Complex topics could be split across multiple webinars to ensure all content is covered. The use of active learning styles can increase attention span [17], and this study found that participants preferred longer sessions with an optimal duration of 60 minutes, which allowed time for questions. 

Throughout the webinar series, the standard Zoom platform was used and there were minimal issues with technology. However, poor internet connection and audio quality can leave participants feeling frustrated. Therefore, speakers, as well as participants, should ensure they have a good internet connection and microphone. In addition, the presence of a session facilitator can allow communication with participants in the event of the speaker losing internet connection or having a device malfunction [18]. 

Social media provides a common channel for healthcare professionals and students to share information and education [19]. Facebook and Instagram are the most common social media platforms used by people in the typical demographic range of medical students and junior doctors [20]. The next most effective method of promotion was direct advertising through medical schools. This is dependent on having a network of contacts at the target universities but could be expanded through contacts from organizations such as the British Orthopaedic Training Association (BOTA), the Association of Surgeons in Training (ASiT), or the British Orthopaedic Association (BOA).

Based on the findings of this study, the lessons learned during the webinar series, and the organisers’ experience of delivering this 12-week online program, the authors produced a “How to Run a Successful Webinar Series for Medical Students” guide to offer educators advice and tips on how to develop and deliver their online teaching effectively.

Limitations of this study include the use of only a post-test analysis. Future studies should use a pre-and post-test design to demonstrate change and improvement in participant knowledge after the course. Also, there was variation in the participants’ level of expertise with differing knowledge levels amongst participants, which could also be evaluated with a pre-and post-test design. 

Descriptive statistics were used to summarize the quantitative data collected from the five-point Likert scales and graphical tools were used to display the results. Future studies should be of a pre-and post-test design, in addition to including the use of five-point Likert scales, to permit statistical analysis to demonstrate any statistically significant change in participants’ knowledge.

The format of the teaching presentations created by the speakers was up to the discretion of the individual speaker with no standardized structure. The BOA undergraduate syllabus was provided to speakers, but there was no peer-review process of the final teaching slides to ensure the syllabus was covered [7]. In addition, the teaching experience of the speakers was unknown, and no teaching training was provided to the speakers. Future virtual teaching courses should consider a peer-review process of the teaching materials and provide medical education training for the speakers to encourage consistency across the webinars.

## Conclusions

The webinar series was well-received and attended, with participants from the UK and abroad. This study revealed key insights into participants' enjoyment of webinars and offered important lessons on organizing a successful webinar series. Interactivity is essential for participant enjoyment and knowledge retention. Additionally, where possible, webinars should be recorded for participants to review in their own time and be accessible to all interested learners. Educators from various academic subjects who will be delivering teaching through online webinars will also benefit from the results of this study.
